# Testing the hypothesis that solvent exchange limits the rates of calcite growth and dissolution†

**DOI:** 10.1039/d4ra00565a

**Published:** 2024-05-14

**Authors:** Nikhil Rampal, Hsiu-Wen Wang, Alexander B. Brady, Jose M. Borreguero, Denys Biriukov, Eugene Mamontov, Andrew G. Stack

**Affiliations:** a Chemical Sciences Division, Oak Ridge National Laboratory Oak Ridge TN 37831 USA stackag@ornl.gov rampaln@ornl.gov wangh3@ornl.gov; b Department of Chemical Engineering, Columbia University New York NY 10027 USA; c Computer Science and Mathematics Division, Oak Ridge National Laboratory Oak Ridge TN 37831 USA; d Central European Institute of Technology, Masaryk University Kamenice 5 625 00 Brno Czech Republic; e Neutron Sciences Division, Oak Ridge National Laboratory Oak Ridge TN 37831 USA

## Abstract

It is established that the rates of solvent exchange at interfaces correlate with the rates of a number of mineral reactions, including growth, dissolution and ion sorption. To test if solvent exchange is limiting these rates, quasi-elastic neutron scattering (QENS) is used here to benchmark classical molecular dynamics (CMD) simulations of water bound to nanoparticulate calcite. Four distributions of solvent exchanges are found with residence times of 8.9 ps for water bound to calcium sites, 14 ps for that bound to carbonate sites and 16.7 and 85.1 ps for two bound waters in a shared calcium-carbonate conformation. By comparing rates and activation energies, it is found that solvent exchange limits reaction rates neither for growth nor dissolution, likely due to the necessity to form intermediate states during ion sorption. However, solvent exchange forms the ceiling for reaction rates and yields insight into more complex reaction pathways.

## Abstract 2

Using neutron scattering dynamics measurements to validate molecular dynamic simulations, it is found that the rates of solvent exchange are not likely to limit the rates of calcite crystal growth and dissolution.

## Graphical abstract

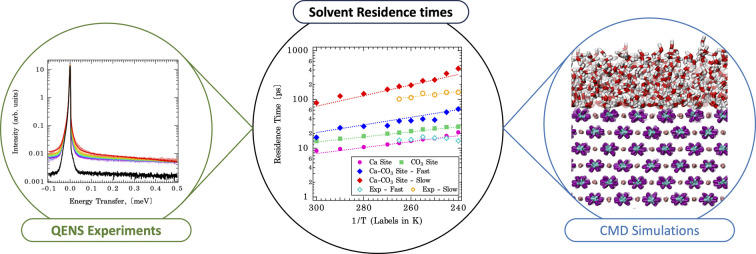

## Introduction

**Fig. 1:**
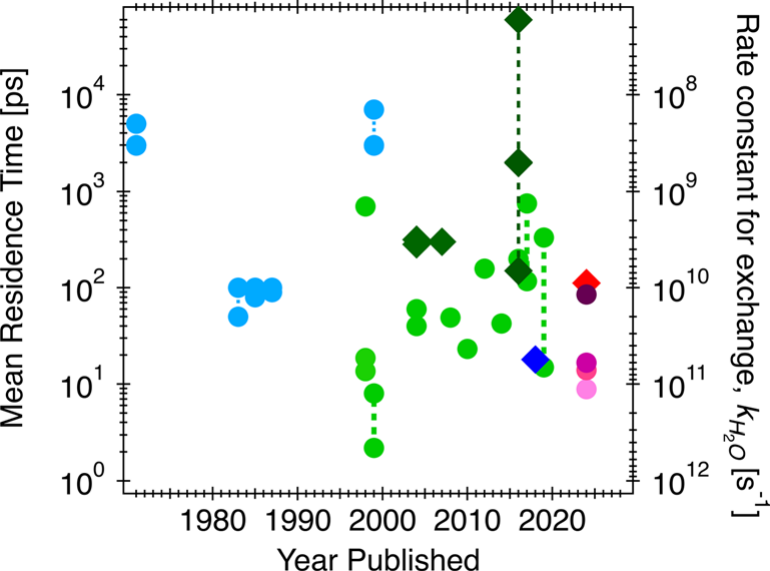
Residence times of water bound to aqueous calcium ions (circles) and on calcite surfaces/nanoparticles (diamonds). Rate constants for water exchange are shown on the right-hand *y*-axis. Light blue circles: experimental estimates of the residence time on aqueous calcium ions; dark blue diamonds: experimental estimates of the residence time of a calcium site on a calcite surface/nanoparticle; light green circles: computational estimates of the residence time on aqueous calcium ions, dark green diamonds: computational estimate of the residence time of water on a calcium site on a calcite surface/nanoparticle. Results from this study (different shades of red/pink): residence time on a calcium ion (red), on a calcium site (light pink), a carbonate site (dark purple), and fast and slow exchanges on a calcium-carbonate site (pink and purple).

Given water's polar nature and its exceptional versatility as a solvent, the rates and mechanisms of water exchange reactions at interfaces plays a pivotal role in a wide range of processes, but frequently find application geochemically in the subsurface and soils (*e.g.*, rates of transformation of CO_2_ into carbonate minerals during sequestration^1^). Solvent exchange is one of the key chemical reactions underlying ion exchange, contaminant adsorption/desorption, and crystal nucleation, growth, and dissolution processes. In fact, Casey^2^ emphasizes the linear relationships established between the logarithms of the dissolution rate and the rates of water exchange about the cation in isostructural series of carbonate,^3^ metal oxide,^4^ and orthosilicate minerals,^5^ as well as ion sorption.^6^ This correlation is sometimes interpreted as causation (discussion below), that the solvent exchange rates on minerals are the rate-limiting reaction for dissolution and related processes. This concept also stems from Eigen–Wilkins,^7,8^ which predicted that no matter the incoming ligands during chelation reactions, the rate-controlling step is the displacement of water molecules bound to cations. Nielson *et al.*^9^ found that the rates of ion attachment during crystal growth are 2–3 orders of magnitude slower than the water solvent exchange rates (*ν*_ion-attachment_ = 10^−3^ × *ν*_dehydration_), attributed to the relationship between solvent exchange rate and rates of ion diffusion to the interface. To test if the correlation between solvent exchange and rates of mineral reactions is indeed a causation, *i.e.*, solvent exchange is the rate limiting step, in this manuscript we first work to resolve the over three orders of magnitude uncertainty in solvent exchange rates for aqueous calcium ion and calcite (CaCO_3_) mineral–water interfaces (Fig. 1). We accomplish this by measuring water exchange rates and mechanisms on calcite nanoparticles through a combination of quasi-elastic neutron scattering (QENS) and classical molecular dynamics (CMD) simulations. QENS is a useful spectroscopic method to probe diffusive motions of hydrogen-bearing species, in this case water, in the time range of ∼1 ps to a few nanoseconds.^10^ Historically it has been used to probe self-diffusion of water^11^ as well as ion diffusion and water exchange.^12,13^ However, at an interface, the large number of different types of water motions can overwhelm analytical fits to the spectra. Thus, here, the overall water dynamics from CMD simulations are fit to the QENS data and then used to interrogate the distributions of rates for specific solvent–exchange reactions and compared to previously-measured rates and activation energies of growth and dissolution. To facilitate comparison and to make the data more readily intuitable, we quantify the average residence times of water molecules to aqueous calcium ion and surface sites on the calcite surface (in ps). This parameter is the reciprocal of the rate constant for water exchange reactions (*e.g.*, in s^−1^),^13^ which in turn can be converted to higher order rate constants to compare to those for growth and dissolution by *e.g.*, including the molar volume of the material.^14^

In Fig. 1, the three orders of magnitude range of previous experimental and computational estimates of the residence times of water for aqueous calcium and calcite surfaces are shown by year of publication. Specifically, using competitive ligand exchange/absorption measurements,^15^ simple analytical model for fitting QENS^12,13,16,17^ and/or proton nuclear magnetic resonance (^1^H-NMR) spectra,^18,19^ experimental data for water residence times on aqueous calcium ion have resulted in estimates ranging from less than 100 picoseconds (ps) to a couple of nanoseconds (ns) (light blue circles in Fig. 1). These observations are sometimes not consistent with computational observations (light green circles in Fig. 1), which range from estimates that are extremely fast, less than 50 ps (usually estimated from first principle simulations^20–24^) to somewhat slower rates less than 800 ps (obtained from classical simulations^25–31^). One such study by Koskamp *et al.*^24^ performed both *ab initio* and classical simulations and estimated the water residence times on aqueous calcium ion ranging from 14.9 to 333 ps. Experimental validation is warranted given the wide range of estimates (also shown in Fig. 1). Also, there is a lack of experimental estimates for solvent exchanges on calcite surfaces, though computational studies have found values in the range of 300 ps^27,28^ and 2 ns,^30^ shown by dark green diamonds in Fig. 1, and for step edges (acute and obtuse) in the range of 0.14 to >60 ns.^30^

In addition to forming a baseline for validating water residence times and understanding reaction kinetics at calcite–water interfaces, calcite was chosen because carbonates are ubiquitous in soils, the Earth's subsurface, and deep-sea sediments^32^ and their weathering plays a role in buffering pH of natural waters. Furthermore, the emissions of greenhouse gases and the detrimental effects on the climate^33^ have stimulated research into energy-efficient methods to sequester and store carbon. Trapping carbon dioxide and converting it to a carbonate mineral has been considered a highly desirable option for long-term storage security,^34^ yet there is a debate over how long precipitation of carbonate minerals might take under different scenarios.^1^ With calcite being such an essential mineral, it is no surprise that it has garnered significant attention and research.

## Methods

### QENS experiment

Calcite nanocrystals (15–40 nm, Sky Springs Nanomaterials) were used with a specific surface area of 19.1 m^2^ g^−1^, as determined from N_2_ BET. These structures were characterized by a PANalytical Empyrean X-ray diffractometer, indicating a pure calcite component (Fig. S1†). The morphology of the calcite nanoparticles was also investigated using transmission electron microscopy (TEM; Hitachi HF2000), which showed typical rhombohedral habit with the {104} cleavage faces (dominated) or with both the {104} and {110} faces (Fig. S2†). Hydration of the dry nanopowders was carried out by allowing powders to equilibrate with laboratory air (approximately 22 °C and 60% relative humidity) for a period of about 24 hours prior to loading into an aluminum sample can for QENS measurements. Thermal gravimetric analysis (TGA) accompanied by mass spectrometric (MS) analysis of the released components was obtained using aliquots of the same hydrated powders used in the QENS experiments. The TGA-MS results are shown in Fig. S3,† and two weight-loss stages were observed at temperatures below 200 °C (Fig. S3b†). In the derivative TG curve, the maxima for these two stages occurred at around 73 and 155 °C, respectively. MS analysis showed predominately water loss at temperatures below 200 °C (0.89 wt%), whereas a continuous weight change consisting of water and CO_2_ loss was observed up to 300 °C (Fig. S3c†). This overlapping mass loss for water and CO_2_ and a lag in between the QENS and TGA-MS measurements created uncertainty in the amount of water adsorbed to the sample.

QENS experiments were performed on the backscattering spectrometer, BASIS, at the Spallation Neutron Source, Oak Ridge National Laboratory. Details of the instrument are described elsewhere.^35^ A scattering momentum transfer range of *Q* = 0.3–1.7 Å^−1^ was used. The *Q*-averaged energy resolution Δ*E* was 0.0035 meV (full-width at half maximum, FWHM). The dynamic range chosen for this experiment was −0.1 to 0.5 meV. Temperatures were ramped down from 265 to 240 K, with an additional measurement at 10 K to obtain the resolution function. Analytical models on QENS line width analyses were carried out using the ICE-MAN workbench^36,37^ for the global *Q*-dependent fit. Briefly, the experimental dynamic structure factor, *S*(*Q*,*E*) is fitted with the functions described as:^38,39^

1 *S*(*Q*,*E*) = *R*(*Q*,*E*) ⊗ [*A*_E_(*Q*)*δ*(*E*) + *A*_n_(*Q*)*L*_n_(*Q*,*E*) + *A*_b_(*Q*)*L*_b_(*Q*,*E*)] + BG

2
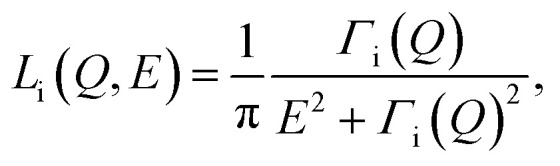
where *R*(*Q*,*E*) is the resolution function of the BASIS spectrometer (measured at 10 K), *δ*(*E*) is the delta function centered at zero energy transfer, *L*_n_(*Q*,*E*) and *L*_b_(*Q*,*E*) are Lorentzian functions, and BG describes a linear background. *A*_E_(*Q*), *A*_n_(*Q*) and *A*_b_(*Q*) are the areas of elastic, narrower Lorentzian, and the wider one, respectively. The functional form of *L*_i_(*Q*,*E*) is by no means unique: it is just one way to extract average relaxation/residence times for the system with complex dynamics. In the global fitting, the power law *Q*-dependence of peak width *Γ*_i_(*Q*) (half-width at half maximum, HWHM) for each relaxation procedure, *i.e.*, *Γ*_i_(*Q*) = *Γ*^*o*^_i_*Q*^*λ*^_i_ is defined by *λ*_i_ and Lorentzian *Γ*^*o*^_i_. These two fit parameters characterize the line width for all *Q* values (as a global feature) at each measured temperature. When plotted as ln *Γ*_i_*vs.* ln *Q*, both *Γ*_n_ (narrow Lorentzian component for slow dynamics) and *Γ*_b_ (broad Lorentzian component for fast dynamics) data follow straight lines with a slope *λ*_i_ and *y*-intercept *Γ*^*o*^_i_. Table S1† summarizes the *Γ*^*o*^_i_ values and *Q*-dependence of the slope *λ*_i_ from the fit, and Fig. S4† illustrates the fitting results. As a side note, this method relies on quantifying the dynamics of hydrogen-bearing species (water) adsorbed to a mineral surface. One might reasonably ask if other species present at the interface, such as dissolved solutes, impurities or sorbed species due to surface charge,^40^ might contribute to the signal. However, first, due to the difference in incoherent scattering between hydrogen and other species, only hydrogen-bearing species will scatter significantly. Second, the influence of water bound to any adsorbed species is expected to be minor. For example, the concentration of calcium and carbonate ions in water in equilibrium with calcite and exposed to the atmosphere is ∼10^−4^ M and 10^−5^ M, respectively. At a bulk water density of 55 000 M, this means there are ∼10^9–10^ water molecules per dissolved ion. The effects of aqueous calcium or carbonate on the total signal will thus be negligible.

### Classical molecular dynamics simulations

**Fig. 2:**
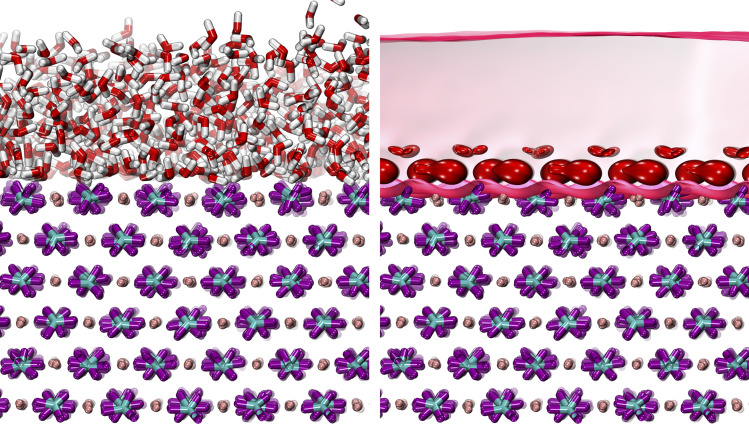
Snapshot of CMD simulation box with 1690 waters (left) and average water density profile over a ten nanosecond (ns) trajectory, showing water distributions on the calcite surface (right). The colors are as follows: calcium – peach, carbon of carbonate – cyan, oxygen of carbonate – purple, and oxygen and hydrogens of water – red and white, respectively. Dark red circles (in the right figure) represent areas of high average oxygen densities (number densities > 0.1 atoms per A^3^), and the light red surface represents the extent of the entire water layer.

Classical Molecular Dynamics (CMD) simulations were performed using the LAMMPS^41^ software package in the canonical ensemble (NVT). The MD simulation box contained a supercell of the calcite {104} surface, created using CrystalMaker® software (Oxford, England). The water molecules were randomly added on top of the surface using Packmol.^42^ The calcite–water supercell was then relaxed using the force fields of Raiteri *et al.*^43^ with an SPC/Fw^44^ water model. The particle–particle particle–mesh solver (pppm) was used for the long-range electrostatic force calculation with a threshold of 1 × 10^−4^. The best estimate from the TGA-MS is that the water surface had ∼1 wt% water adsorbed on it (Fig. S3†). Modeling roughly 1 wt% water content on a 4782.62 Å^2^ calcite slab yields to 845 waters on a calcite {104} surface. Due to uncertainty in the amount of sorbed water on the surface due to overlapping TGA-MS mass losses of water and CO_2_, three different water coverages were tested: the 845 water estimate that corresponds to 1 wt%, this estimate was doubled to 1690 waters, and doubled again to 3380 waters to model the QENS spectra. The system containing 1690 waters is illustrated in Fig. 2, and for others, see Fig. S5 and S6.†

First, each of these supercells were equilibrated for one nanosecond, followed by a production run of 10 ns with a timestep of 1 fs. The trajectories were then harvested every 100 steps (0.1 ps) and stored for subsequent fitting to QENS spectra and calculating the residence times of water. Next, fitting to the QENS data was initiated by calculating a two-time point self-correlation function to derive the intermediate self-incoherent structure factor *I*(*Q*,*t*). From this, the dynamic structure factor *S*(*Q*,*E*) was calculated by taking the Fourier transform of *I*(*Q*,*t*). To model the QENS data, the correlation between the simulated and experimental QENS spectra at each value of the momentum transfer *Q* is described by eqn (3):

3 *S*(*Q*,*E*) = *A*·*R*(*Q*,*E*) ⊗ [*xδ*(*E* − *E*_0_) + (1 − *x*)*S*_MD_(*E* − *E*_0_)] + BG

where *S*_MD_(*E*) is the model structure factor obtained from CMD simulations, *R*(*Q*,*E*) is the resolution function (measured at 10 K), and *δ*(*E*) is the delta function for the elastic peak, centered at zero energy transfer. *x* is the fraction of the elastic-incoherent scattering, *A* is a scaling factor between a fit and the experimental spectra, and BG is a linear background. Parameter *E*_0_ allows for systematic deviation of the experimental elastic line from its theoretical location, *i.e.*, *E* = 0. The *xδ*(*E* − *E*_0_) term accounts for the missing contributions in CMD simulation trajectories but are present in the experiment, *e.g.*, the signal generated from the calcite matrix, the sample holder and the solvent coherent scattering within the instrument resolution width. This methodology has been explained in detail in Stack *et al.*^45^

### Calculation of residence times

Residence times, *τ*, of sorbed water on the calcite surface are calculated by a direct counting method. Each water molecule is tracked and assigned to one of four observed states over the simulation trajectory. These four states are water molecules bound to either the Ca-site, CO_3_-site, a shared Ca–CO_3_ site, or not bound to the calcite surfaces at all (*i.e.*, unbound states). These states were first observed by examining probability isosurfaces (845 waters *vs.* 1690 waters), shown in Fig. 2 and S8.† The state to which a water molecule belongs is determined using a distance cutoff between the water and each surface site. The minima of the first and second shells from the radial distribution function (details in the Section S7†) are used for these cutoffs. For the Ca-site, if the oxygen of water (O_w_) is within 3 Å of the calcium atom, it is considered in a bound state. On the other hand, if the hydrogen of water (H_w_) is within 2.3 Å of oxygens on the carbonate (O_CO_3__), it is considered bound to the CO_3_-site. There is a third possibility that the water can be bound to two surface atoms, both Ca and CO_3_ (*i.e.*, to Ca–O_w_ and H_w_–O_CO_3__ simultaneously). This is the case when the water molecule simultaneously satisfies both the cutoff criteria. If none of these cutoffs are satisfied, a water molecule is considered as in the unbound state. In addition to these distance criteria, a 2 ps cutoff is implemented to distinguish a successful exchange attempt from an unsuccessful one; that is, if the water leaves a specific surface site for more than 2 ps, it is considered as successfully exchanged to either a different site or to an unbound configuration. Similarly, a water molecule must be associated with a specific site for more than 2 ps to be successfully bound to that site. A 2 ps time tolerance is implemented because the time-dependent transmission coefficients using the reactive flux method usually reach a plateau in 1–2 ps, indicating a successful exchange.^46–48^ With a 1 ps cutoff, a significant number of 1 ps exchanges have been found previously, but without a corresponding distribution of exchanges (*i.e.*, a large number of precisely 1 ps exchanges with low numbers of exchanges of similar residence times), suggesting this is an artifact that represents unsuccessful exchanges.^45^ Lastly, a histogram of residence times was made using a log-normal plot and the multi-peak fitting routine in the Igor Pro software (Lake Oswego, OR, USA).

### Deconvolution of QENS spectra

Our previous work successfully demonstrated a method for decomposing model water populations to the observed QENS intensities, which allows quantitative validation of the solvent structure and exchange dynamics predicted from the CMD simulation.^45^ Following a similar methodology, different water populations (bound and unbound) are defined on their initial and final configurations relative to the surface sites. This results in three types of water exchange dynamical motions, which are bound-to-bound (BB), bound-to-unbound or unbound-to-bound (BU/UB), and unbound-to-unbound (UU). These combinations (BB/BU/UB/UU) thus signify the bound states of water at time *t*_initial_ and *t*_initial_ + *t*, *e.g.*, bound-to-bound (BB) state is when the water molecule is bound to the surface at *t*_initial_ and at *t*_initial_ + *t*. Generally speaking, BB motions dominate at the low-energy region of the QENS spectra, signifying longer-time dynamics. In contrast, UU motions contribute to the higher-energy regions, representing shorter-time dynamics.

## Results and discussion

As described above, the first step in deriving residence times for water on the calcite surface is to measure the diffusional motions of water at the calcite {104}–water interface experimentally. In part to confirm that the diffusional motions within the dynamic range of the QENS instrument are similar magnitudes to the solvent exchange dynamics, we first fit an analytical model (eqn (1)). The measured QENS data is fit using two Lorentzian peaks over the *Q* range of 0.3–1.7 Å^−1^, indicating a weak *Q*-dependence of peak width (Fig. S4†). The *Q*-dependence of the fitted slope *λ*_n_ (narrow Lorentzian component) goes from 0.29(2) at 265 K to 0.26(3) at 240 K, and the fitted slope *λ*_b_ (broad Lorentzian component) goes from 0.34(2) at 265 K to 0.19(5) at 240 K (Table S1†). A meaningful comparison can be made based on the *λ* value. For *λ* = 2 the standard Brownian diffusion is recovered, while *λ* < 2 indicates a localized diffusive character. Since the observed value *λ* ≪ 2 for both slow (*λ*_n_) and fast (*λ*_b_) components, it suggests that water-bound to calcite surfaces are confined and possess a localized diffusive character, as expected for solvent exchange reactions.

**Fig. 3:**
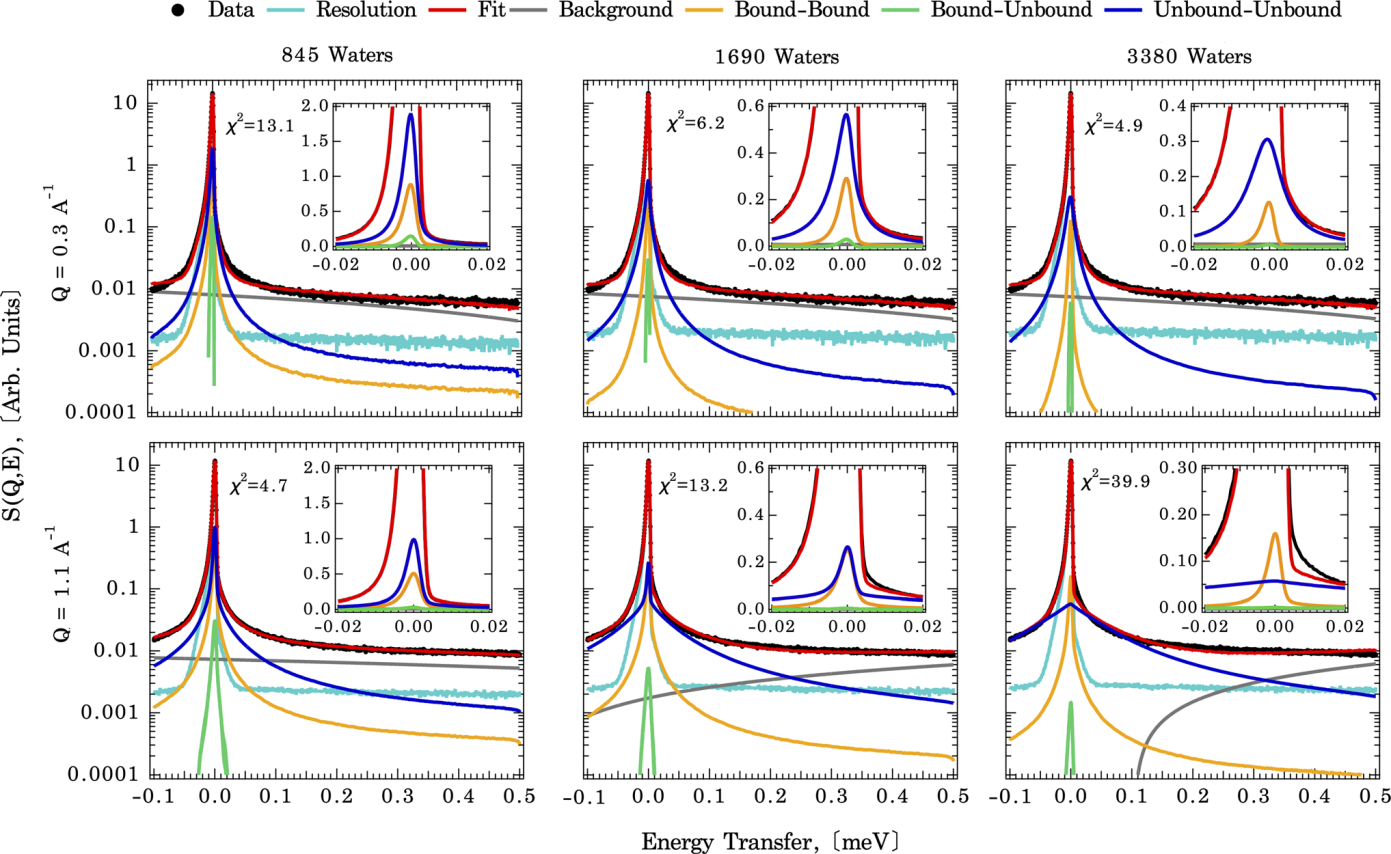
QENS experimental spectra (black points) indicating the dynamics of the system, with computed CMD fit (red line) for three water coverages (left to right) at *Q* = 0.3 Å^−1^ (top row) and *Q* = 1.1 Å^−1^ (bottom row) at 265 K, deconvoluted to contain three types of water motions between states (initial to final) in the system: bound-to-bound (BB) (yellow), averaged bound-to-unbound (BU) and unbound-to-bound(UB) (green); and unbound-to-unbound (UU) (blue). The cyan and dark grey lines represent the resolution function and the background.

Fig. 3 illustrates direct comparisons between the experimentally measured QENS spectra (at 265 K and *Q* = 0.3 and 1.1 Å^−1^) of calcite nanoparticles with a few layers of sorbed water to those computed from the CMD trajectories. Note: since the *Q*-dependence of QENS peak width is weak, we only compute dynamics up to *Q* = 1.1 Å^−1^ to save some computational times. The QENS spectra predicted by the CMD simulations using three different water coverages are overlaid on the experimental data, along with their deconvolution into contributions from bound and unbound states. The fits are qualitatively similar for all three water coverages (845, 1690, and 3350 waters) at their respective *Q* values, with subtle differences in the energy transfer region of −0.02 to 0.02 meV, *i.e.*, close to the elastic peak (*E* = 0 meV).

A closer examination of the fit results shown in Fig. 3 suggests that the differences in the simulated QENS spectra from different water coverages arise from the contribution of UU and BB water motions in the system. When the water coverage increases, the relative contribution to the total intensities from UU water motions increases (blue curve), as expected, and BB water motions decrease (yellow curve). Our approach to the deconvolution of the QENS spectra serves an important purpose: it confirms that the solvent exchange reactions (*i.e.*, the BB signal) on the mineral surface contributes significantly to the observed QENS intensities. Thus, once validated, the CMD simulations will provide accurate information about water exchange dynamics, especially in predicting QENS signals at low *Q* values, which are dominated by longer and slower water diffusional/exchange motions.

**Fig. 4:**
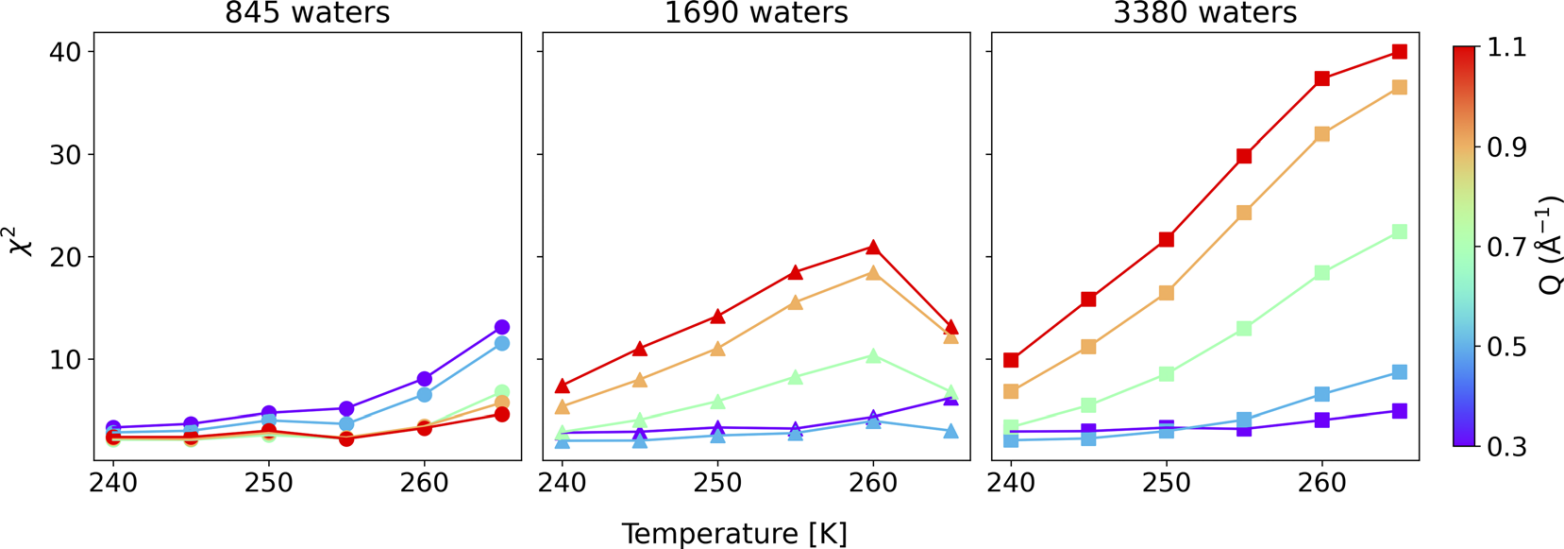
*χ* ^2^ Goodness-of-fit weighted by the experimental error for the CMD fit to experimental QENS data as a function of temperature over all three water coverages. Different water coverages have different symbols (circles, triangles and squares for 845, 1690 and 3380 waters, respectively), whereas measurements at different momentum transfer values are differentiated by color (red is 1.1 Å^−1^, orange is 0.9 Å^−1^, green is 0.7 Å^−1^, Å^−1^, purple is 0.3 Å^−1^).

To compare the quality of the fits, the *χ*^2^ (goodness-of-fit) values are calculated and weighted by the experimental error, shown in Fig. 4 (and Fig. S7†). At low *Q* values, all the water coverages match the experimental QENS data. As the water coverage increases, the fits progressively worsen (indicated by an increase in *χ*^*2*^ values), especially at higher *Q* values. Because scattering from high *Q* regions is mainly characterized by faster (short-time) water motions (*e.g.*, water rotational motions), it is clear that the higher water coverage model (3380 waters) overestimates the signal from these motions, likely due to it containing an excess contribution from unbound waters relative to the experiment. Unfortunately, distinguishing the correct water coverage model on the studied sample system is not straightforward because with increasing water coverage, the quality of the fits showed strong *Q*-dependent character, and the *χ*^*2*^ values cannot be used as the only qualifier. For this study, the goal is to understand the solvent exchanges. Thus, a better fit at lower *Q* values is prioritized rather than a better fit at higher *Q* values. This rules out the 845-water model due to its poorer fit to the low *Q* data. It suggests that the simulation may not have sufficient bound water molecules to characterize local diffusive motions (BB) observed in the experiments correctly. This argument is consistent with previous work, which showed that observed dynamics at mineral–water interfaces is progressively slower as water coverage is diminished.^49^ The 3380-water model can also be ruled out because the above-mentioned poor fits due to an excess of unbound water (UU). Therefore, moving forward, we select the 1690-water model as the closest water coverage to the experimental data. We reiterate that there does not seem to be an ideal water content, which suggests the possibility that the force field might not reproduce all of the dynamics in the system equally well. As a parameterized method, this is a fairly common outcome for CMD, where optimizing one type of structure, dynamics, or energetics may lead to a worse agreement with other properties.^50^ A detailed discussion of the possible origins of discrepancies is discussed below.

**Fig. 5:**
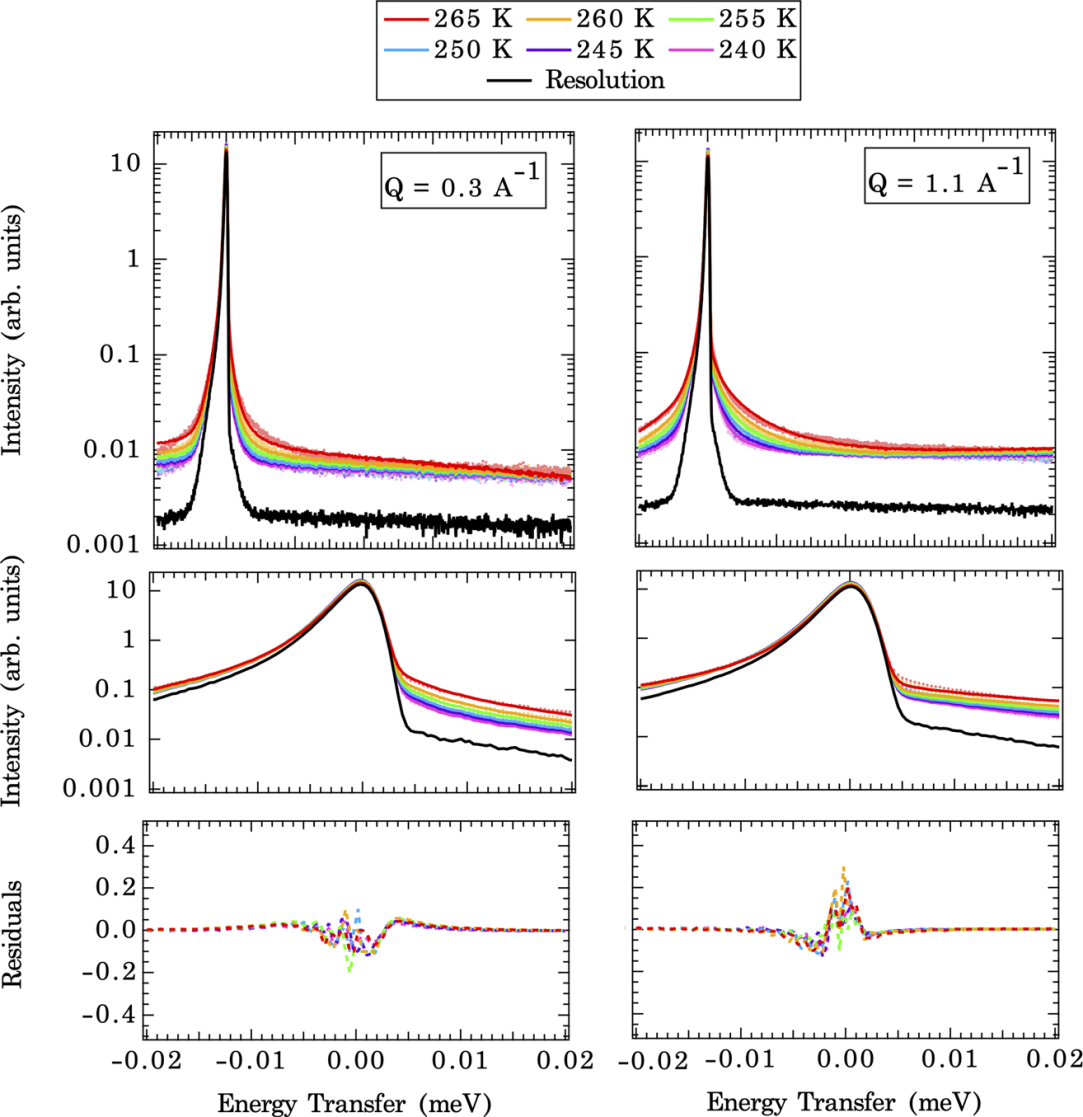
First row: QENS spectra (dashed lines) with computed CMD fit (solid line) for 1690 waters at *Q* = 0.3 Å^−1^ (left) and *Q* = 1.1 Å^−1^ (right) for all the temperatures ranging from 240 K to 265 K (colors are shown in legend). Second row: QENS spectra zoomed in on the elastic peak in the *Q* range of −0.02 meV to 0.02 meV. Third row: residual function (QENS experiment – CMD fit).

The temperature-dependent QENS spectra fits at two selected *Q* values (CMD simulated end members) are shown in Fig. 5 (upper panel). A closer view of the fits near the elastic peak (*E* = 0 meV) region and the corresponding residual signals are displayed in the middle and bottom panels of Fig. 5. The broadening of QENS line width with increasing temperatures is expected since the mobility of water molecules increases with temperature. The implemented force field does a good job of replicating the experimental QENS spectra over the temperature range studied. The inconsistencies, especially at the shoulder of the elastic peak (±0.005 meV), between computed QENS spectra and experimental observations can arise from several sources. First, we are modeling a pristine {104} calcite surface, which is an idealized approximation since the calcite nanoparticles in the QENS experiment may have different morphologies/terminations and numerous defects with unknown contributions to the total signal. This approximation is reasonable since the calcite {104} surface is the most energetically favorable termination compared to other surface terminations: {0001}, {1010},{101̄1}, and {112̄0}.^51^ This same approach has been adopted by Stack *et al.*,^45^ in analyzing QENS spectra of barite nanoparticles using the {001} barite surface as the representative structure in CMD simulations. However, the actual particles do contain a mixture of {104} and {110} surfaces (Fig. S2†), so the {110} surfaces could contribute to the error. While the termination of the {110} is expected to be similar to the {104}, a different orientation of the surface carbonates may create some differences in the dynamics of the system. A second source of error could arise from the force field used in the CMD simulations. We have utilized Raiteri *et al.*’s^43^ force field, which is benchmarked to both the bulk properties of calcite (free energy of dissolution and lattice parameters), the solution structure (ion–water distances, ion pairing free energies, and coordination numbers), and the water densities at the interface.^52^ Our previous work^50,53–55^ suggests that these “formal charge” type force fields tend to overestimate the extent of the ordering of the solvation shell structures and the attraction of ions/water to surfaces.^10,53,56,57^ Third, experimental uncertainties come with the QENS measurements, such as intensities generated from the calcite matrix, the sample holder, and the solvent coherent scattering within the instrument resolution width. The linear background and elastic peak fitting in eqn (3) account for the scattering of many of these effects, but how completely the fitting procedure corrects these is not known. Given all of these uncertainties, a readily identifiable solution cannot be implemented to improve the fit quality. Thus, we conclude that the model is close to the local optimum regarding the fit quality and now proceed with interrogating solvent exchange rates.

**Fig. 6:**
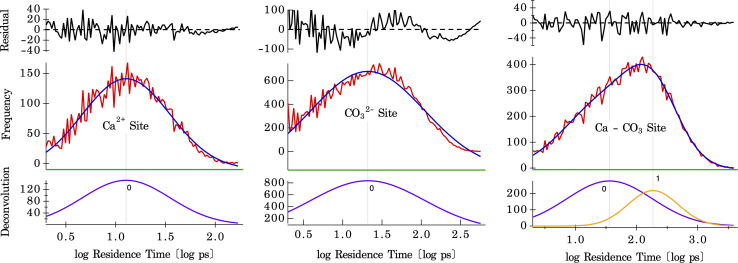
Residence time distributions of water exchanges occurring at specific sites (Ca, CO_3,_ and shared Ca–CO_3_ site) on the Calcite {104} surface at 265 K. Each column represents a different surface site with the first row representing the residual of the distribution of water exchanges (red curve, second row) to the log-normal fits (blue curve, second row) and its respective deconvolutions (purple curve + orange curve, bottom row). At 265 K, the Ca-site represents a single distribution at 1.11 log ps = 12.7 ps, the CO_3_-site has a single distribution at 1.32 log ps = 20.9 ps, and the shared Ca–CO_3_ site shows a bimodal distribution that is comprised of a fast component 1.56 log ps = 36.1 ps and a slow component at 2.27 log ps = 185.2 ps.

The residence times of water are calculated on three different surface sites present on the calcite surface, shown by histograms plotted in Fig. 6. These histograms were fit using a single Gaussian peak for water bound to Ca and CO_3_ sites and two Gaussian peaks for the shared Ca–CO_3_ site. The difference between one and two Gaussian fits for the CO_3_ site, and the shared Ca–CO_3_ site is shown in Fig. S10.† These results (shown in Fig. S11†), along with the temperature dependence of the residence times, are tabulated in Table 1. For water bound to the Ca-site, a mean residence time of *τ* = 8.9 ps (at 300 K) is found, whereas, on the CO_3_-site, it is *τ* = 14.0 ps (at 300 K) (there may, in fact, be two distributions of solvent exchange on this site, based on the residuals in Fig. 6 center). There are two distinct distributions for water in the shared Ca–CO_3_ configuration (shown in Fig. 6), with mean residence times of *τ* = 16.7 ps and 85.1 ps (at 300 K). The latter is the slowest solvent exchange observed on the calcite surface, indicating that the shared conformation offers a measure of stability (maximum lifetime) for water molecules bound to calcite surfaces. The faster exchange observed here, with a residence time of 16.7 ps, is close to that of water residence times on the CO_3_-site, which suggests a lateral exchange reaction between nearby binding sites. Such intra-surface water exchange between neighbouring surface sites has been observed on barite–water interfaces.^37^ The fact that it is observed in calcite–water interfaces also suggests that lateral water exchanges may be a common feature of mineral–water interfaces. Since it has no counterpart in solvent exchanges on aqueous ions, this unique property will confer differing reaction mechanisms for reactivity between surfaces and solutes. For example, a hypothesis for the effect of solute ions on quartz dissolution is that electrolyte ion adsorption stimulates an intra-surface hydrogen bonding conformation that is the precursor to dissolution.^58^

**Table 1:** Mean residence times computed from CMD simulations for the temperature range of 240 K to 300 K for surface sites (top panel) and the activation energies in kJ mol^−1^ associated with each surface site (bottom panel). The shared Ca–CO_3_ site is comprised of the fast exchange (first column) and the slow exchange (second column)

Temperature [K]	Residence Times [ps]			
	Ca-site	CO_3_-site	Shared Ca–CO_3_ site	
300	8.9	14.0	16.7	85.1
290	9.7	15.7	26.5	116.9
280	10.5	17.7	28.0	130.4
270	11.9	19.8	28.9	160.4
265	12.7	20.9	36.1	185.2
260	14.1	22.4	36.6	194.9
255	15.1	24.3	39.8	240.7
250	15.8	25.5	38.0	251.5
245	17.9	27.2	54.7	338.8
240	21.2	27.7	63.6	428.8
Arrhenius activation energy [kJ mol^−1^] ± 1				
Ca-site			8.3	
CO_3_-site			7.1	
Shared Ca–CO_3_ site (fast)			11.1	
Shared Ca–CO_3_ site (slow)			14.8	

**Fig. 7:**
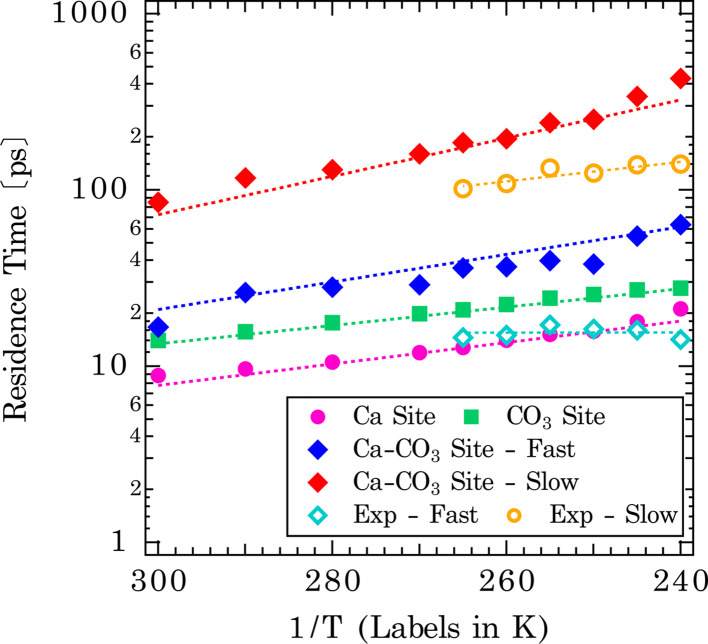
Average residence time distributions of water on calcite {104} surface as a function of temperature. 3 distinct sites with 4 distributions are observed: fast exchanges from the monodentate Ca and CO_3_-site and a slower solvent exchange from the bidentate Ca–CO_3_ site. An empirical fit to the experimental QENS data (orange and light blue) using two Lorentzian distributions is also shown.

The extracted mean residence times of solvent exchanges are shown in the Arrhenius-type plot in Fig. 7 (shown by dotted lines for each type of exchange). The derived activation energies are tabulated in the bottom section of Table 1. The singular distributions of exchanges on Ca and CO_3_ sites have shorter residence times and naturally lower activation barriers for exchanges (Ca-site: *E*_a_ = 8.3 kJ mol^−1^, CO_3_-site: *E*_a_ = 7.1 kJ mol^−1^). These represent more facile exchanges to and from surface/bulk. For the shared Ca–CO_3_ conformation, higher activation energies and slower exchange rates are observed: Ca– CO_3_ site (*E*_a_ = 11.1 and 14.8 kJ mol^−1^, for fast and slow exchanges, respectively). This is rationalized as water molecules bound to two surface sites will have a longer residence time. For comparison, the temperature dependence of the residence times, obtained from the Lorentzian *Γ*^*o*^_i_, as *τ*_i_ = *ℏ*/*Γ*^*o*^_i_, is also shown in Fig. 7 and summarized in Table S2† (*ℏ* is the reduced Planck constant). It should be noted that we did not make assumptions concerning the origin of particular components for these analytical fits. Both fast (*τ*_b_) and slow (*τ*_n_) components probed by BASIS exhibit an Arrhenius-type temperature dependence (Fig. 7). It is evident that the analytical model fits cannot distinguish, for example, water bound to the Ca- *vs.* to CO_3_-sites due to similar residence times and would have difficulty capturing such fine variations. However, the similar time scales from the Lorentzian fits offer further evidence that the QENS signal is sensitive to the solvent exchange processes at mineral–water interfaces. Compared to water molecules trapped in amorphous calcium carbonate, the residence time determined from one Lorentzian model fit to the QENS spectra is about 18 ps at 300 K and 21 ps at 250 K.^59^ Here, we observed a comparable result in residence times between water molecules bound to the Ca- or CO_3_-sites on calcite surfaces and water molecules in the amorphous calcium carbonate network.

Given the vast range of previously reported residence times (Fig. 1), an important extrapolation is to use the same force field to calculate water residence times on the aqueous form of the ions. Our estimate of the solvent exchange on the aqueous calcium ion using the Raiteri *et al.*^43^ force field is 112 ± 4 ps. Compared to other estimates (100 ps and 105 ps)^23,43^ using the same force field, these estimates can be treated as within the expected variation between implementations and analysis. Compared to other computational estimates from different classical/empirical force field methods, those estimates range from 5 ps to 800 ps (Fig. 1).^25–31^ The large variability in these results demonstrates that a predicted solvent exchange rate highly depends on the force field's functional form and parameterization. Some of these force fields are parametrized to the solvation structure and some to the energetics of ions (hydration/solvation energies). On the other hand, when comparing CMD to quantum chemical/first principle type calculations (DFT/AIMD/CPMD/QM-MM),^20–24^ the estimated residence times for the latter tend to be much shorter (15–60 ps). One of the main reasons for much shorter residence times is that the sampling time is extremely limited because it is computationally more expensive to sample longer timescales using the higher level of theory. This problem is exacerbated since solvent exchanges take log-normal distributions, and first principle calculations cannot capture longer residence times within the simulation timeframe. In comparison, experimental estimates of solvent exchanges (on calcium ions) are much slower (>500 ps) than any computational estimates.^12,13,16^ Our current QENS measurements show relatively faster dynamics at both calcite–water interfaces and aqueous calcium ions than experimental observations. One potential complicating factor is that the QENS instrument (BASIS^35^) used here is sensitive to a specific dynamic range, that is, ∼6 ps–2 ns so may not be sensitive to slower exchanges. In contrast, other experimental instruments and techniques sample a different dynamic range so may not be comprehensive either. Other types of experimental estimates of solvent exchanges, such as competitive ligand exchange, for instance, may offer only a distorted view of the rate of water exchange since a different reaction is actually being measured (*i.e.*, exchanging a ligand for a water molecule rather than a self exchange of one water for another). Similarly, NMR estimates of residence times are typically nanoseconds or longer.^18^ Nonetheless, since our analytical model fit results in characteristic times matching the solvent exchange rates predicted from the CMD (Fig. 7), it suggests that our CMD model is predicting accurate solvent exchange rates over the spectrometer's dynamic range. Subsequently, the residence time of water on aqueous calcium ion (112 ± 4 ps) can be considered a robust estimate.

The implications of this study for the reactivity of calcium carbonate minerals are that the dehydration of the pristine calcite surface is not the rate-limiting step for the dissolution of calcium carbonate minerals,^60^ nor likely their growth. That is, the processes responsible for limiting the nucleation rate correlate with the water exchange rates but are not likely caused by it (*e.g.*, Casey^2^ demonstrated this correlation for dissolution). Though it has been hypothesized that solvent exchange is the rate-limiting step compared to ion attachment/diffusion, our results support the idea that solvent exchange is not rate-limiting. The evidence for this assertion is as follows: first, the activation energies for solvent exchange on calcite–water interfaces are far smaller than that of dissolution. Measured activation energies for the steady-state calcite dissolution that are far from equilibrium are in the range of 33–42 kJ mol^−1^.^3,61^ These measurements are much larger than the 7–15 kJ mol^−1^ activation energies for solvent exchange on different sites measured here (Table 1). However, one study at high temperature and close to equilibrium did find activation energies for steady-state monomolecular step retreat of 25 ± 6 and 14 ± 13 kJ mol^−1^ for obtuse and acute orientations of steps, respectively.^62^ The low values measured there were interpreted as representing the influence of the back-reaction on the activation energy (*i.e.*, ion attachment influencing dissolution). Estimates for calcite growth are in a similar range as for dissolution, 33–39 kJ mol^−1^,^63,64^ consistent with computational estimates of ion attachment from metadynamics simulations using this force field.^65^ (Despite similar range of activation energies, the rate constants vary between growth and dissolution so their absolute rates are different, despite similar temperature dependencies.^3,61–64^) Thus, we conclude that activation energies, which are strongly linked to the reaction mechanism, differ between solvent exchange and rates of dissolution and growth. In contrast to the crystalline phase, dissolution of amorphous calcium carbonate occurs much more quickly, but activation energies are not known, so it remains possible that solvent exchange limits the rate of dissolution of amorphous calcium carbonate.^59,66,67^

A second line of evidence beyond the activation energies is that one can calculate a first-order rate constant for solvent exchange by taking the reciprocal of the residence times measured here as a first-order rate constant and comparing that to rate constants for ion attachment derived from fits to monomolecular step advance during crystal growth (such models have not been fit to dissolution data unfortunately). Thus, the rate constant at 300 K for solvent exchange on the calcium site is 1.1 × 10^11^ s^−1^, carbonate is 7.1 × 10^10^ s^−1^, and the shared calcium–carbonate site's two populations of exchanges are 6.0 × 10^10^ s^−1^ and 1.2 × 10^10^ s^−1^. Our most recent fitted first-order rate constants for ion attachment during calcite growth are *k*_Ca_ = 5.6 ± 0.2 × 10^6^ s^−1^, *k*_CO_3__ = 3.0 ± 0.1 × 10^7^ s^−1^.^68–70^ These are 4 and 3.4 orders of magnitude smaller than the rate constants for solvent exchange on the aqueous calcium and carbonate sites, respectively, and 3-4 orders of magnitude smaller than the solvent exchanges on the shared Ca–CO_3_ site. The rate constants for attachment are of similar magnitude to other process-based model fits, *e.g.*, *k*_Ca_ = 2 × 10^6^ and *k*_CO_3__ = 1 × 10^7^ s^−1^.^71^ (The reported second order rate constant values have been divided by the molar volume of calcite, 0.03693 L mol^−1^, to obtain first order rate constant units after.^14^) While previous work has suggested that solvent exchange ought to be two to three orders of magnitude faster than ion attachment, the reason for that range has been attributed to ion diffusion to a kink site.^9,24^ This view is difficult to reconcile with computational simulations of ion attachment and detachment on surfaces, which show similar activation energies as measured experimentally but without incorporating ion diffusion.^65,72^ In contrast, activation energies for ion diffusion will clearly be driven by rates of solvent exchange, as cited above (Table 1), also in ref. 10. In addition, solvent exchange rates are much smaller than those of growth and dissolution.

Taking both lines of evidence, it indicates that solvent exchange alone is not rate limiting for ion attachment during growth, but there is a significant contribution from the endothermic penalty for the formation of intermediate states where the ion sheds one or more waters of hydration (disruption of ion hydration shell). This penalty is not fully compensated by the exothermic formation of new bonds to the surface. Such states are characteristically found in metadynamics simulations of ion attachment and detachment to mineral surfaces^65,72^ as well as unexpected shifts in the rates of ligand exchange reactions of poly-oxo-metallates with cation substitutions.^2,73^

## Conclusions

The dynamics of water at calcite–water interfaces were measured using QENS experiments and used to validate diffusional dynamics in CMD simulations. Validating the CMD simulations by the experimental data allows us to use the CMD to robustly resolve the individual water motions and diffusions at the surface. Simulations are benchmarked to different waters in the CMD simulations to reproduce the most appropriate comparison to QENS measurements. The QENS spectra are deconvoluted into different bound/unbound water components, confirming that solvent exchanges significantly contribute to the diffusional dynamics measured in the QENS experiments. Three conformations of water bound to the surface originate from water being associated with either one of the two singular conformations on Ca or CO_3_ surface sites or a shared conformation of a single water molecule bound to both the Ca and CO_3_ surface sites simultaneously. The singular conformations each show a single distribution of residence times, which follow a log-normal distribution with an average of 9 ps for water bound to the Ca-site and 14 ps for water on the CO_3_ site at room temperature. The shared Ca–CO_3_ site has a bimodal log-normal distribution of residence times, comprising a fast exchange with an average residence time of 17 ps and a slow exchange of 85 ps at room temperature. This force field also predicts a residence time of water on an aqueous calcium ion of 112 ± 4 ps, consistent with previous work using the same parameters. The activation energies for dissolution (33–42 kJ mol^−1^) and growth (33–39 kJ mol^−1^) are larger than the activation energies for solvent exchange (7–15 kJ mol^−1^). Similarly, rate constants for attachment of calcium and carbonate to calcite monomolecular step edges during crystal growth are 3–4 orders of magnitude smaller than rate constants for solvent exchange measured here. These findings strongly suggests that solvent exchange is one process within a multi-step growth and dissolution process, but is not rate limiting directly (*i.e.*, the impressive correlation between dissolution and growth rates and solvent exchange is not a causation).

## Author contributions

Nikhil Rampal: investigation, methodology, visualization, formal analysis, writing – review & editing. Hsiu-Wen Wang: investigation, supervision, formal analysis, writing – review & editing. Alexander B. Brady: methodology, writing – review & editing. Jose M. Borreguero: methodology, software, writing – review & editing. Denys Biriukov: methodology, writing – review & editing. Eugene Mamontov: investigation, formal analysis, resources. Andrew G. Stack: conceptualization, investigation, writing – review & editing, project administration, funding acquisition, supervision.

## Conflicts of interest

There are no conflicts to declare.

## Supplementary Material

RA-014-D4RA00565A-s001
