# Supplementary material for: Testing the hypothesis that solvent exchange limits the rates of calcite growth and dissolution
Source: RSC Adv. 2024 May 14;14(22):15743–54. doi: 10.1039/d4ra00565a (PMC11093105; doi:10.1039/d4ra00565a)
Supplement: RA-014-D4RA00565A-s001 [file RA-014-D4RA00565A-s001.pdf]

Electronic Supplementary Material (ESI) for RSC Advances.
This journal is © The Royal Society of Chemistry 2024

# Supplementary Information: Testing the Hypothesis that Solvent Exchange Limits the Rates of Calcite Growth and Dissolution

Nikhil Rampal[1,2]*, Hsiu-Wen Wang[1]*, Alexander B. Brady[1], Jose M. Borreguero[3], Denys Biriukov[4], Eugene Mamontov[5], Andrew G. Stack[1]*.

1. Chemical Sciences Division, Oak Ridge National Laboratory, Oak Ridge, TN 37831, USA
2. Department of Chemical Engineering, Columbia University, New York, NY 10027, USA
3. Computer Science and Mathematics Division, Oak Ridge National Laboratory, Oak Ridge, TN 37831, USA
4. Central European Institute of Technology, Masaryk University, Kamenice 5, 625 00 Brno, Czech Republic
5. Neutron Sciences Division, Oak Ridge National Laboratory, Oak Ridge, TN 37831, USA

# 1. Powder X-ray diffraction of calcite nanocrystals

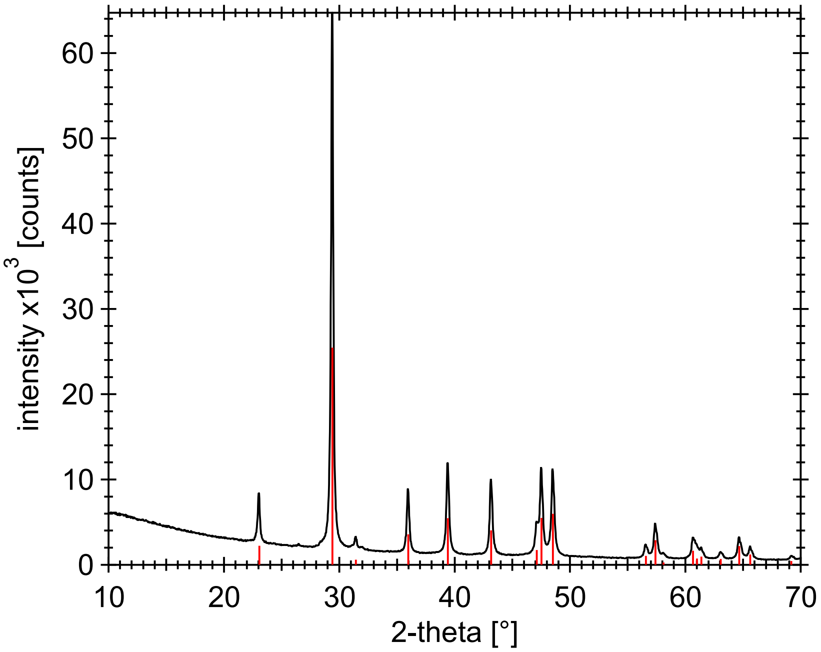


*Figure S1. Powder X-ray diffraction pattern (XRD, black line) of calcite nanocrystals (15-40 nm in diameter). The red straight lines show standard diffraction positions and relative intensities of bulk calcite powder peak. Method: Sample powders were lightly pressed onto a zero-background quartz plate and were characterized using a PANalytical Empyrean X-ray diffractometer (Cu-Kα radiation) with patterns recorded from 5° to 90° 2θ and counting for 0.185 sec at each 0.026° 2θ step, i.e., 10 min measurements.*

## 2. TEM images of calcite nanocrystals

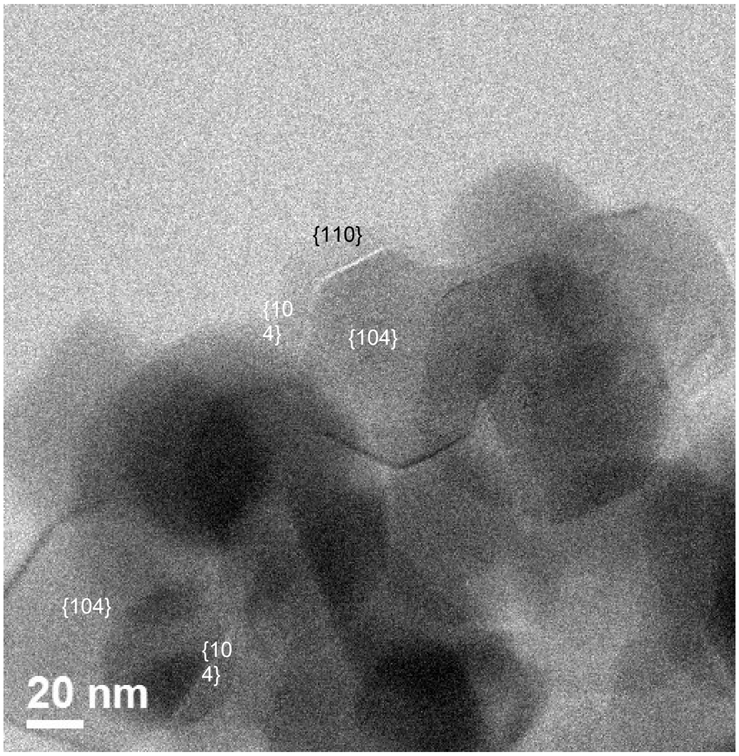


*Figure S2. TEM images of calcite nanocrystals showing typical calcite habit.*

# 3. TGA MS

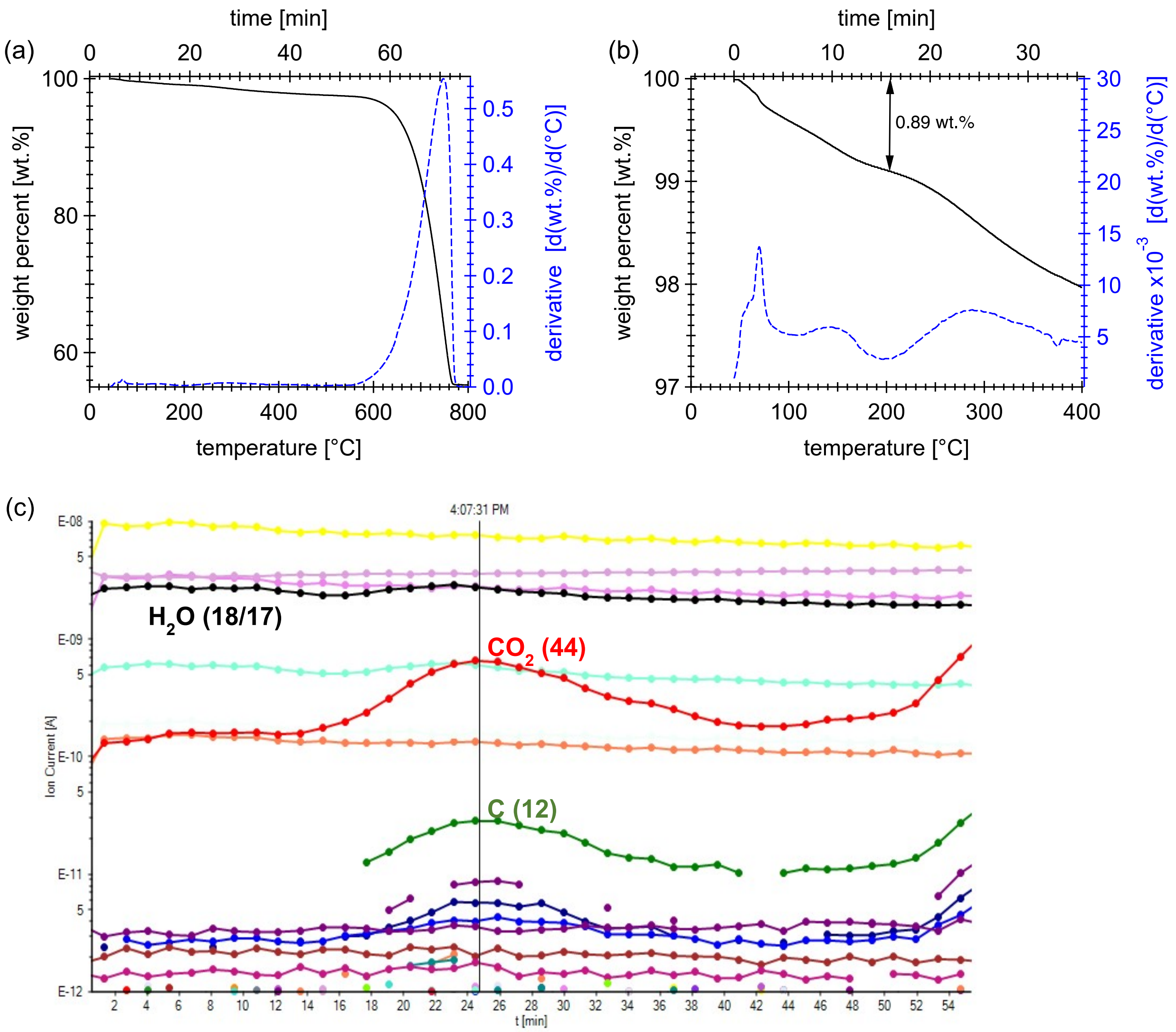


*Figure S3. Thermogravimetric analysis (TGA)-mass spectrometry (MS) data for calcite nanocrystals exposed to laboratory air. (a) TG weight curve (black) as a function of temperature. Thermal decomposition of calcite takes place between 700-800 °C (as a main weight loss feature). (b) A closer look at lower temperature region. Below 200 °C, the derivative TG curve (blue) shows the maxima at two temperature stages, ~73 and ~155°C, respectively. (c) MS results, the y-axis is m/z values detected evolving from the sample as a function of time. Water (m/z's 17 and 18) was seen to be the dominant volatile species during both weight loss events (at temperatures below 200 °C or 16 min). However, a continuous weight change consisting of water and CO2 loss was observed up to 300 °C, indicating that both water and CO2 are the volatile species on these surfaces. Method: The sample was analyzed by a TA Q5000 TGA interfaced to a Pfeiffer Thermostar mass spectrometer by means of a heated capillary transfer line. In this system, the sample was heated from*

*room temperature to 800 °C at 10 °C/min. The capillary transfer line was heated to 195 °C. The Thermostar unit is based on a quadrupole design, and the masses scanned ranged from 1-200 amu. The sample gas from the TGA was ionized at 70 eV. The experiment took place in argon with a flow rate of 35 mL/min.*

## 4. Analytical model fit to the QENS data.

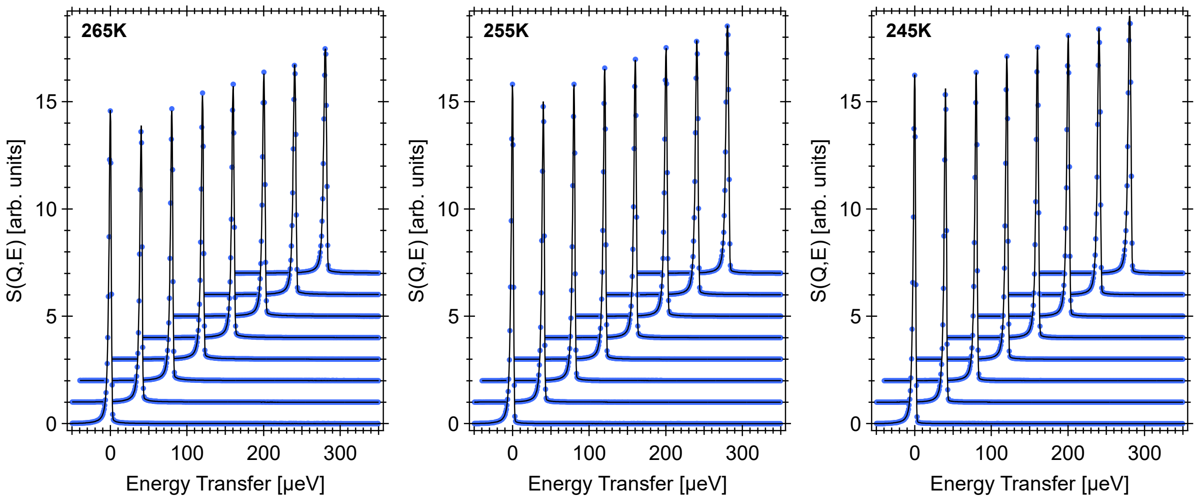


*Figure S4. QENS spectra line width analysis for calcite nanoparticles with a few layers of sorbed water, showing fitted results at 265, 255, and 245 K. Each plot displays the eight different Q values in a sequence of 0.3, 0.5, 0.7, 0.9, 1.1, 1.3, 1.5, and 1.7 Å$^{-1}$ (front to back). Experimental points are shown as blue circles with error bars smaller than the symbols. Black lines are the best fits based on the global Q-dependent fitting procedure. Similar quality of fits was obtained for the 260, 250, and 240 K data and are not shown here.*

**Table S1**. The *y*-intercept HWHM $\Gamma_i^o$ (in μeV) and the slope $\lambda_i$ from the global Q-dependent fit.

| *T* [K] | slow: $\Gamma_n^o$ / $\lambda_n$ | fast: $\Gamma_b^o$ / $\lambda_b$ |
|---|---|---|
| 265 | 6.47(5) / 0.29(2) | 45.2(4) / 0.34(2) |
| 260 | 6.05(6) / 0.27(2) | 43.8(5) / 0.23(3) |
| 255 | 4.94(5) / 0.26(2) | 38.4(5) / 0.18(3) |
| 250 | 5.28(7) / 0.26(3) | 40.5(8) / 0.14(4) |
| 245 | 4.73(6) / 0.30(3) | 41.1(9) / 0.21(5) |
| 240 | 4.69(7) / 0.26(3) | 46.4(9) / 0.19(5) |
| The standard deviation values are shown in parentheses. | | |

**Table S2**. The residence times $\tau_i$ (in ps) and activation energies (kJ/mol) were determined from the fits.

| *T* [K] | slow: $\tau_n$ | fast: $\tau_b$ |
|---|---|---|
| 265 | 101(1) | 15.6(1) |
| 260 | 109(1) | 15.0(2) |
| 255 | 133(1) | 17.1(2) |
| 250 | 125(2) | 16.2(3) |
| 245 | 139(2) | 16.0(4) |
| 240 | 140(2) | 14.2(4) |
| $E_a$ | 9.3(3) | 1.9(4) |
| The standard deviation values are shown in parentheses. | | |

## 5. Calcite water coverages

Snapshots of CMD simulations of different water coverages on the calcite {104} surface are shown below:

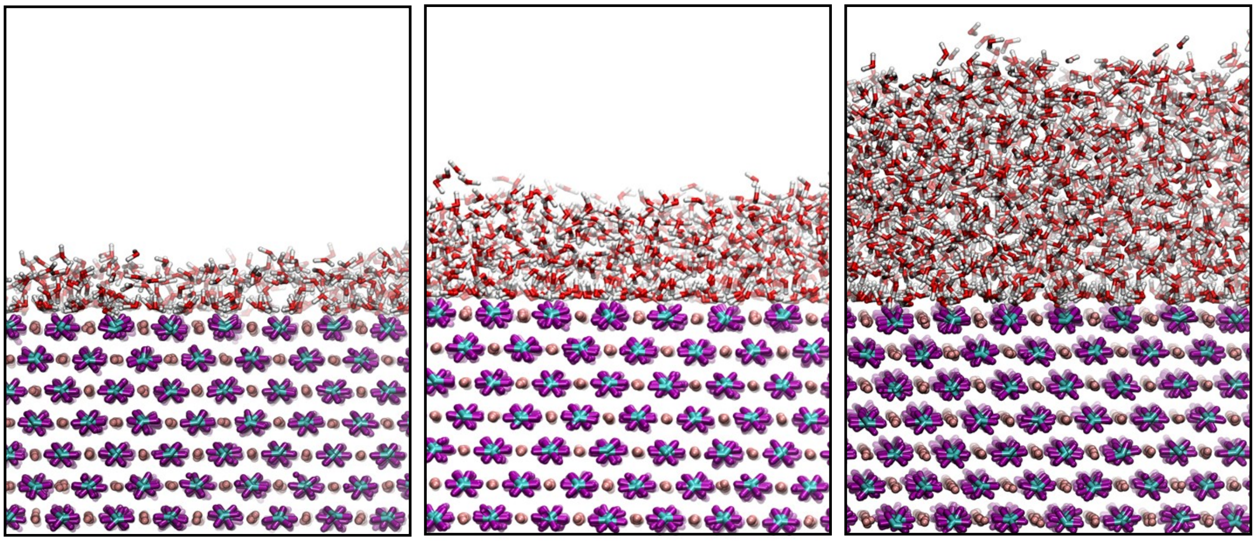

*Figure S5. CMD snapshots of 845 vs. 1690 vs. 3380 water coverages on the calcite {104} surface*

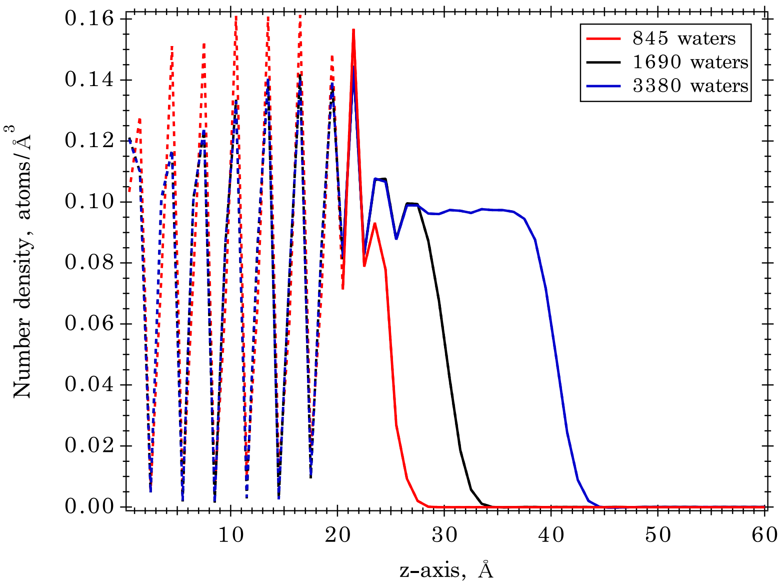


*Figure S6. Number densities of water as a function of distance from the surface for all water coverages*

The respective chi-squared ($\chi^2$) values for each water coverage (845 waters vs. 1690 waters vs. 3380 waters) are shown below as a function of the Q values over the range of temperatures.

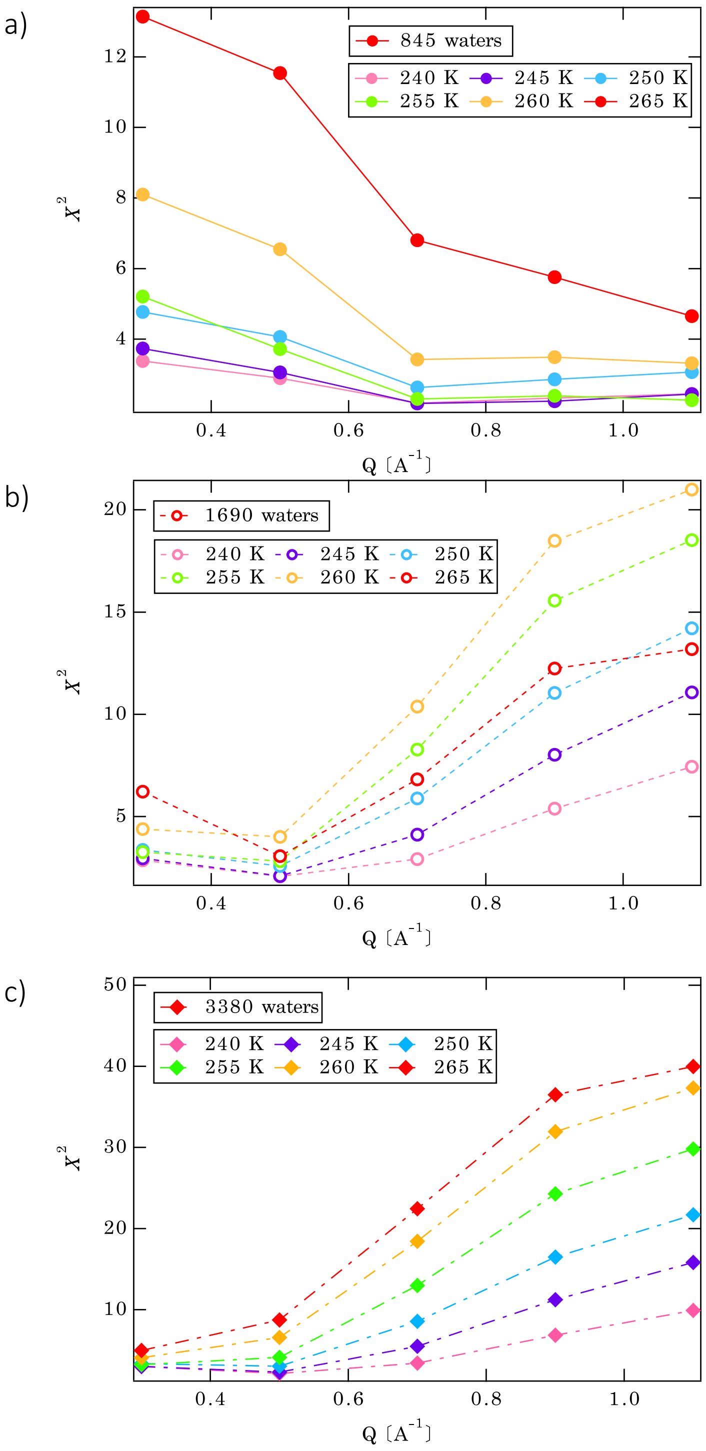


*Figure S7. Chi-squared ($\chi^2$) values of each water coverage as a function of q values over a range of temperatures from 240 to 265 K and for different water coverages, a) 845 waters, b) 1690 waters, and c) 3380 waters.*

## 6. 3D density plots

To visually identify the various states of water molecules: bound to either the Ca-site, $CO_3$-site, a joint Ca-$CO_3$ site, or not directly bound to the mineral surface at all (unbound state), the isosurfaces (3D density plots) of the immediate water bound to the calcite surface is shown below. It is observed that there are single sites for the 845-water coverage (light blue). In contrast, for 1690 waters (orange), multiple water probabilities signify multiple exchanges corresponding to multiple surface sites. The first site is above the calcium ion, and the second site is above the carbonate site (pink and green, respectively). In addition, those two sites are probable, corresponding to the Ca – $CO_3$ sites.

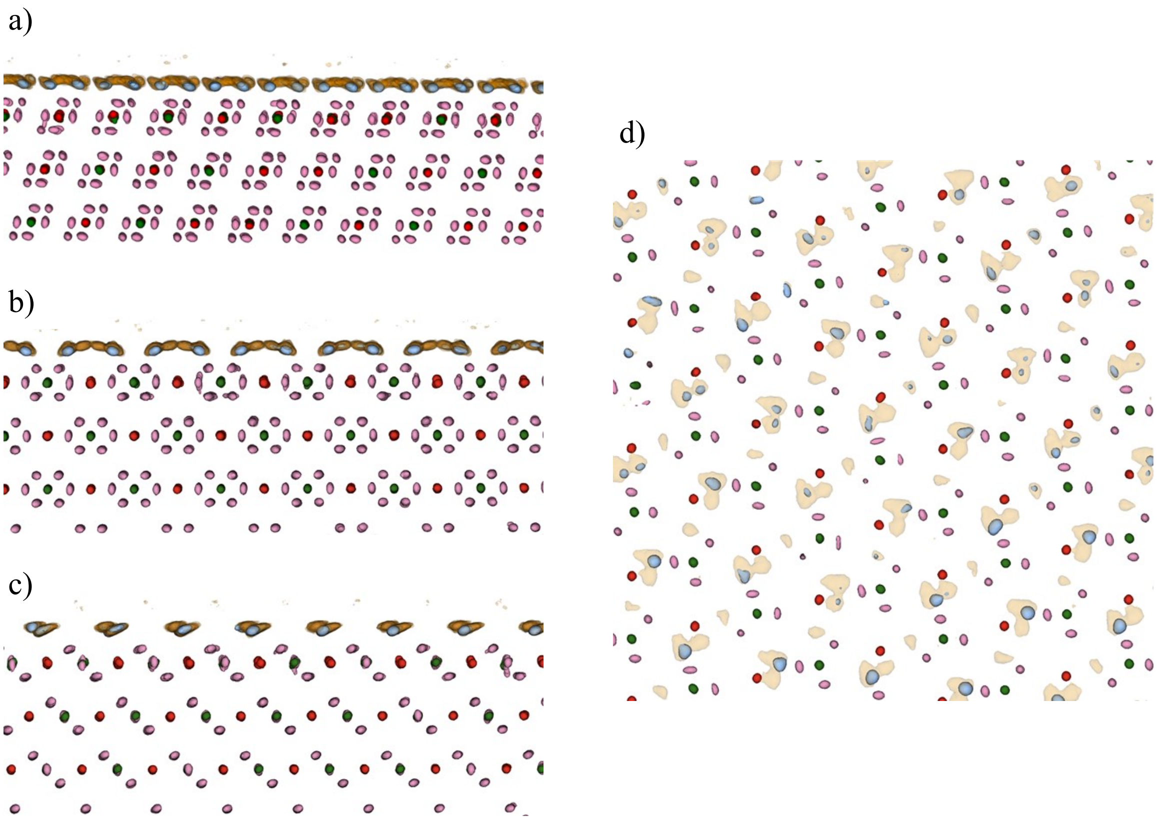


*Figure S8. Probability isosurfaces of water oxygens ($O_w$) on calcite surface. The atoms correspond to calcium (red), carbon and oxygen of carbonate (green and pink, respectively), and the oxygens of water corresponding to 845 water coverage (light blue) and 1690 waters (orange). Figure a), b) and c) are isosurfaces viewed along different planes of the surface. Figure d) is the top view of the water directly associated to the surface.*

## 7. Radial distribution functions

To define the threshold of whether a water molecule is bound or unbound to the surface site, the minima of the first and second shells from the radial distribution are used for this cutoff. For the calcium site, if the oxygen on water ($O_w$) is within 3 Å of the calcite (Ca), it is considered bound. If the hydrogen on water ($H_w$) is within 2.3 Å of oxygen on the carbonate ($O_{CO3}$), it is considered bound to the carbonate site. There is a third possibility that the water can be bound to a bidentate site (Ca-$O_w$ and $H_w$- $O_{CO3}$ simultaneously) for which both the cutoff criteria are satisfied at the same time.

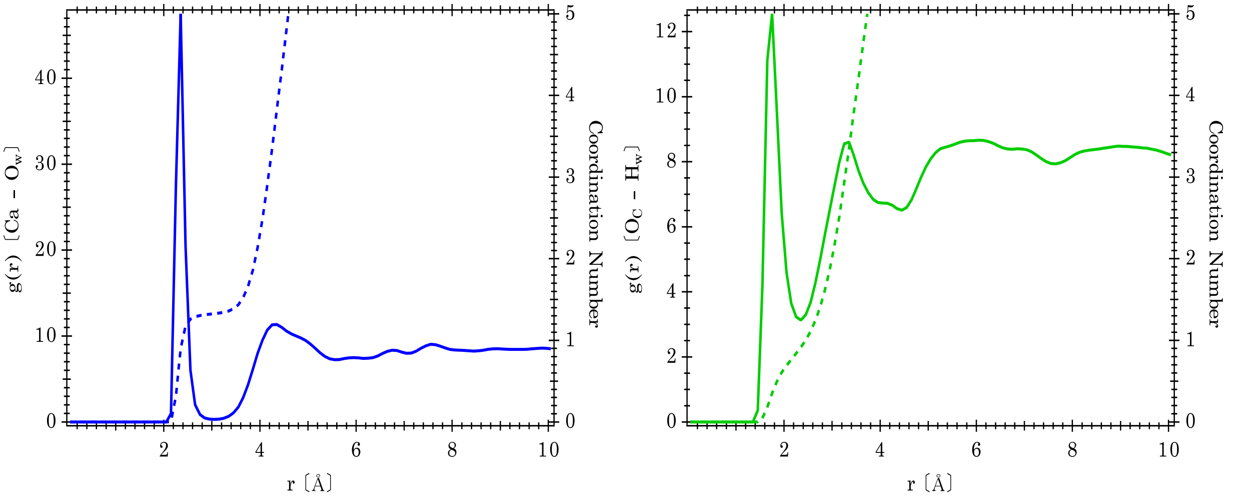


*Figure S9. Radial distribution functions (solid line) and coordination numbers (dashed line) of Ca (left-blue) and $CO_3$ (right – green) sites on calcite {104} surface with water.*

## 8. One vs two Gaussian peaks fit for the $CO_3$ site and shared Ca-$CO_3$ site

The solvent exchange distribution was fit to one and two gaussian models for a) $CO_3$ site and b) shared Ca-$CO_3$ site. Row c) The ratio of the $\chi^2$ values calculated for a single-gaussian fit to a double-gaussian model is compared for all the temperature values. If $\chi^2$ = 1, there is perfect agreement between one and two gaussian fits. It is observed that there isn't a large difference in fitting the CMD-derived solvent distribution to one or two gaussian fits for the $CO_3$ site (values are relatively close to 1). Whereas the double-gaussian model represents a better fit (~ 3 times better) than the single-gaussian fit for solvent exchange on the shared Ca-$CO_3$ site, clearly emphasizing the need to use a two-gaussian model.

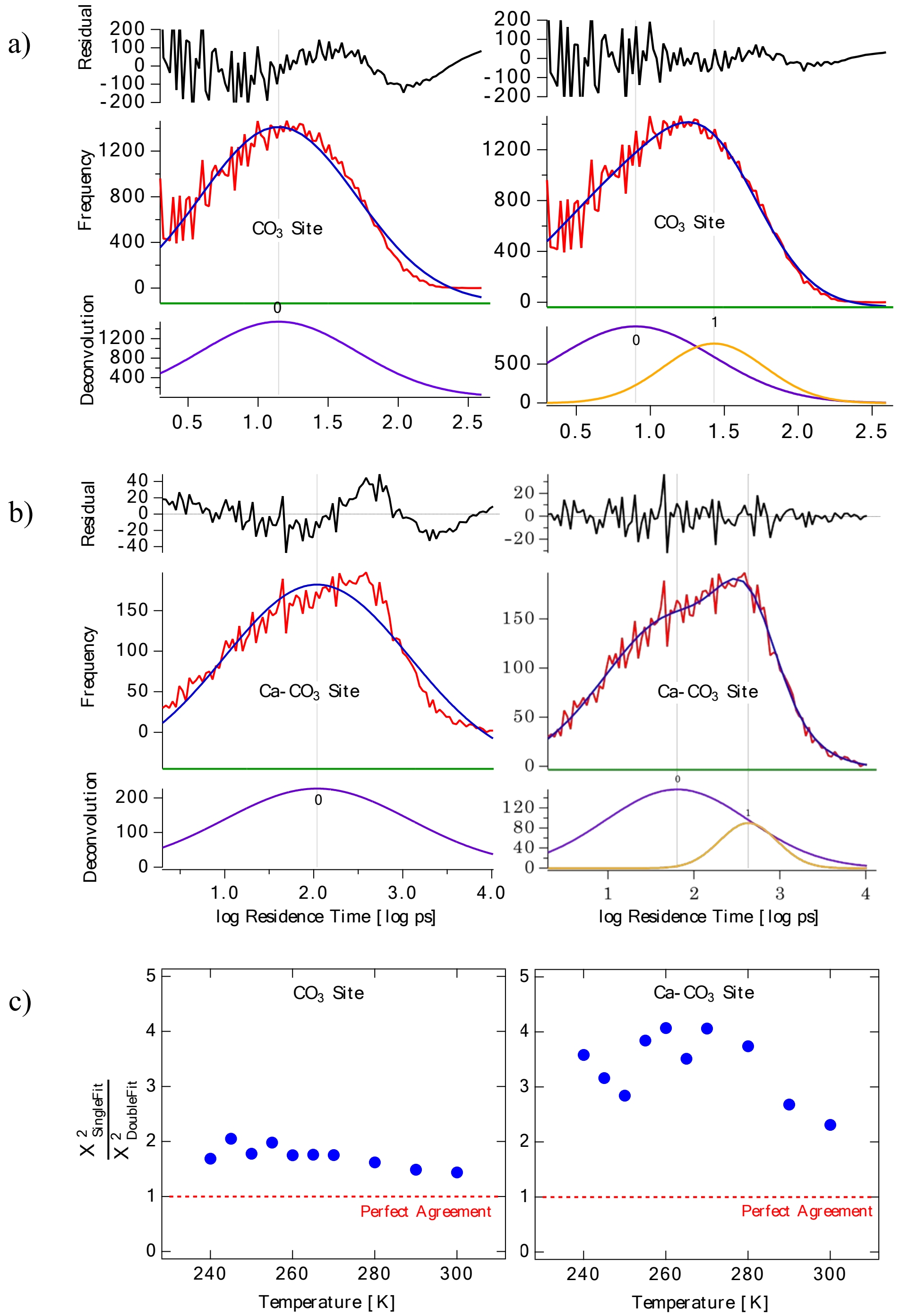


*Figure S10. Residence time distributions of water exchanges occurring at the a) $CO_3$ site and the b) shared Ca-$CO_3$ site on the Calcite {104} surface. Each column represents a different surface site with the first row representing the residual of the distribution of water exchanges (red curve, second row) to the log-normal fits (blue curve, second row) and its respective deconvolutions (orange + purple, bottom row). c) distribution of $\chi 2$ value over the temperature ranges. The value on the y-axis is the ratio of $\chi 2$ value single-gaussian fit to double-gaussian model is compared for the temperature values and site configurations.*

9. Peak fit for distributions of solvent exchanges at Ca Site, $CO_3$ site, and shared Ca-$CO_3$ site

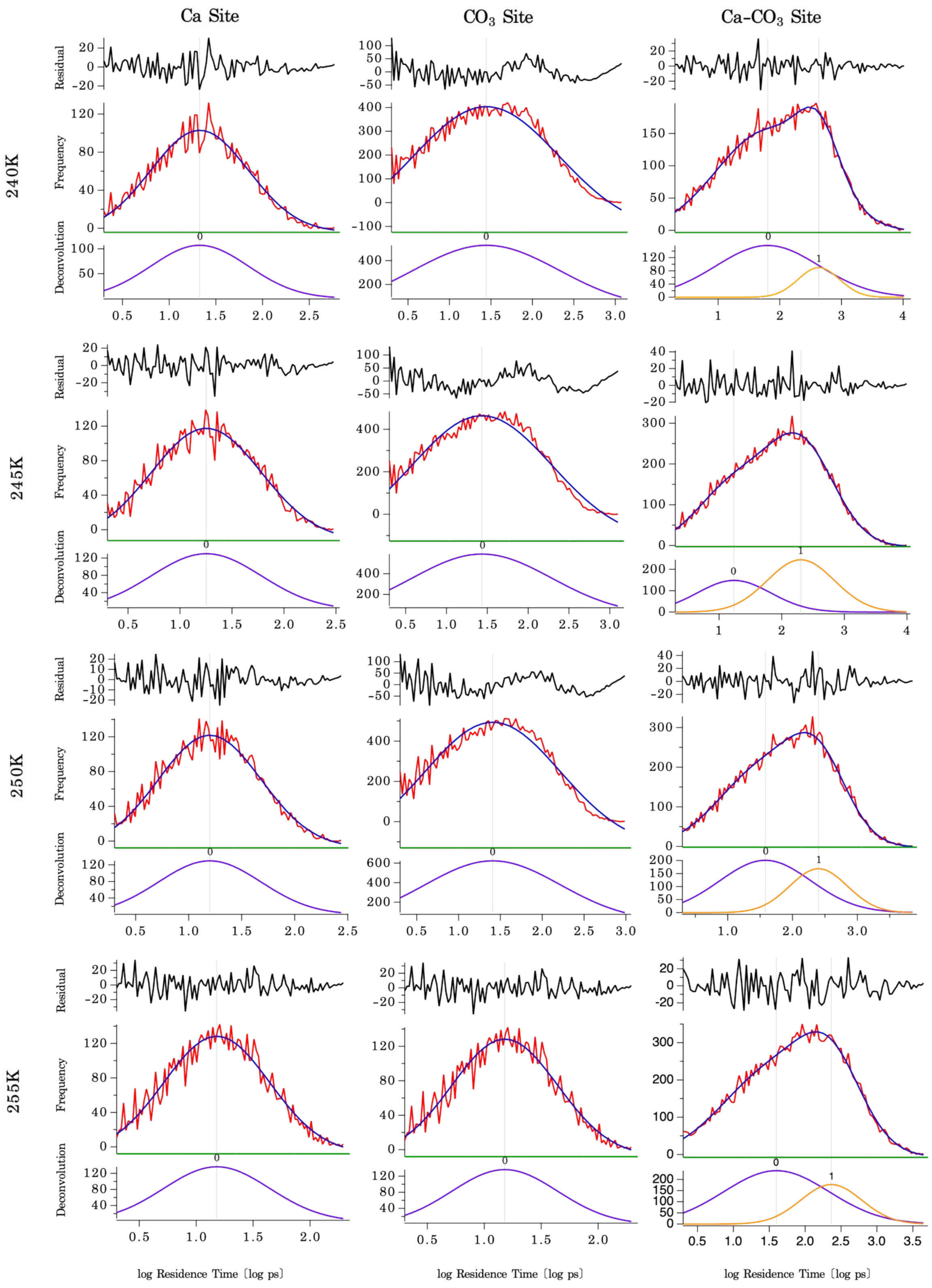

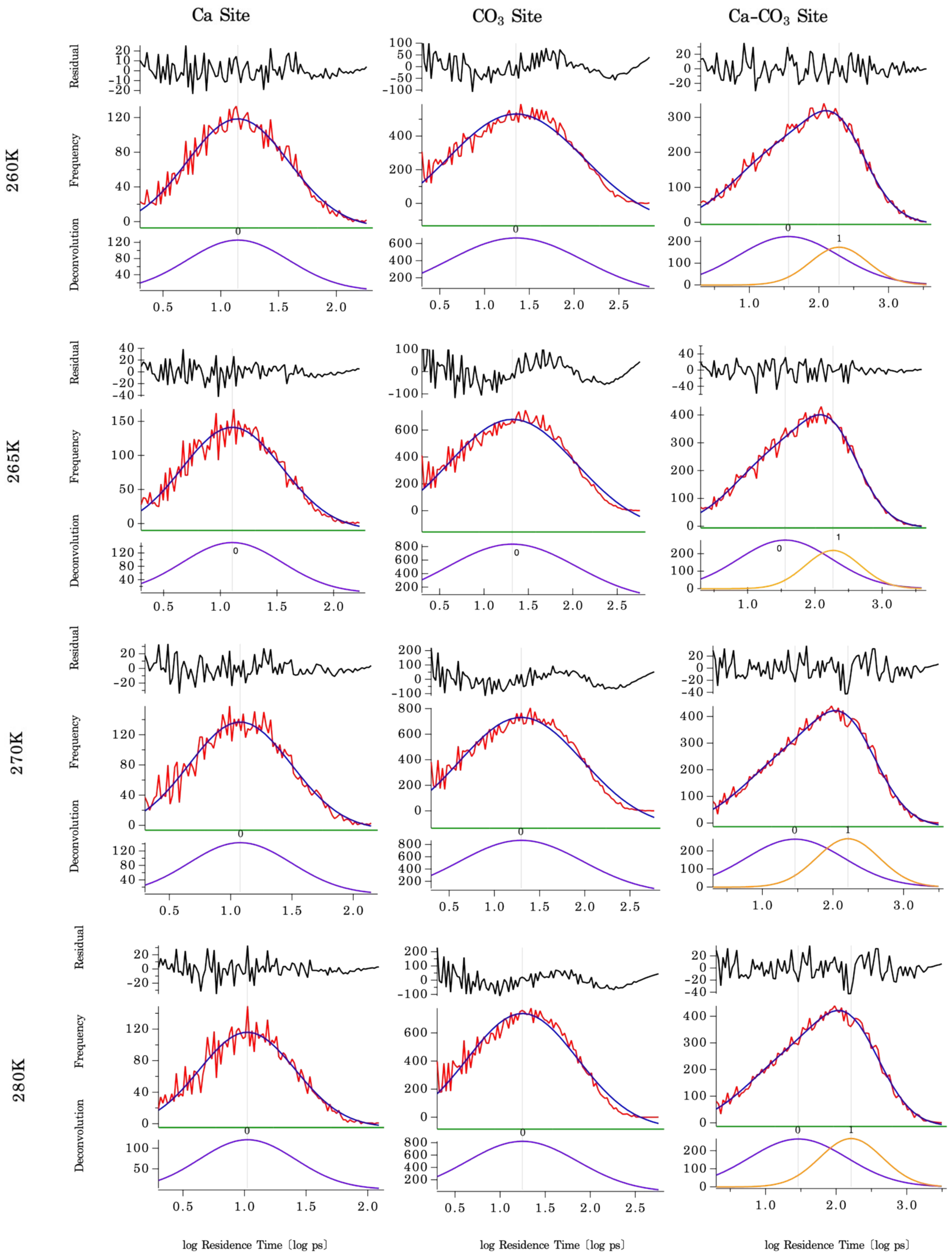
Ca Site
CO$_3$ Site
Ca-CO$_3$ Site
260K
265K
270K
280K
Residual
Frequency
Deconvolution
log Residence Time [log ps]
log Residence Time [log ps]
log Residence Time [log ps]

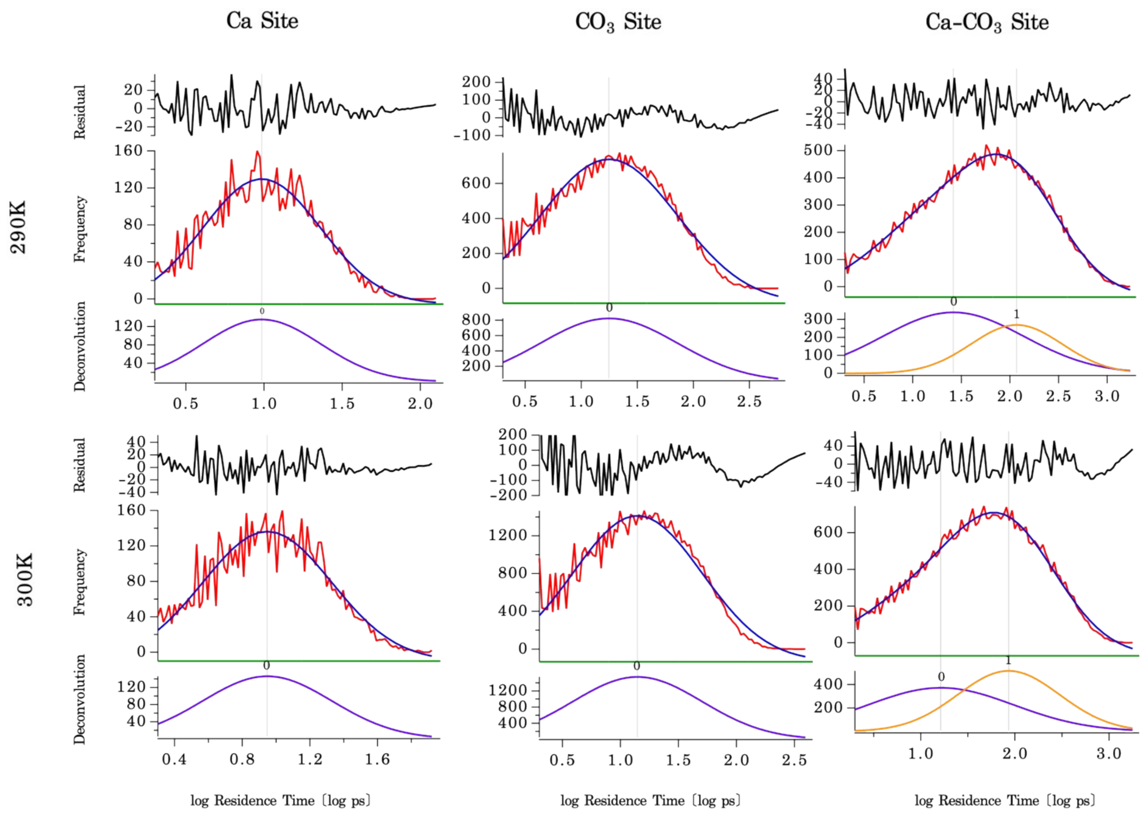


*Figure S11. Residence time distributions of water exchanges occurring at a Ca-Site (left panel), $CO_3$-site (center panel) and Ca-$CO_3$ site (right panel) on the Calcite {104} surface for the temperature range 240K – 300K. Each column represents a different surface site with the first row representing the residual of the distribution of water exchanges (red curve, second row) to the log-normal fits (blue curve, second row) and its respective deconvolutions (purple + orange, bottom row).*
